# Metabolomic alterations in the blood plasma of older adults with mild cognitive impairment and Alzheimer’s disease (from the Nakayama Study)

**DOI:** 10.1038/s41598-022-19670-y

**Published:** 2022-09-08

**Authors:** Tomoki Ozaki, Yuta Yoshino, Ayumi Tachibana, Hideaki Shimizu, Takaaki Mori, Tomohiko Nakayama, Kazuaki Mawatari, Shusuke Numata, Jun-ichi Iga, Akira Takahashi, Tetsuro Ohmori, Shu-ichi Ueno

**Affiliations:** 1 Department of Neuropsychiatry, Molecules and Function, Ehime University Graduate School of Medicine, Shitsukawa, Toon, Ehime 791-0295 Japan; 2 Department of Psychiatry, Institute of Biomedical Science, Tokushima University Graduate School, 3-18-15 Kuramoto-cho, Tokushima, 770-8503 Japan; 3 Department of Preventive Environment and Nutrition, Institute of Biomedical Sciences, Tokushima University Graduate School, 3-18-15 Kuramoto-cho, Tokushima, 770-8503 Japan

**Keywords:** Neuroscience, Biomarkers, Medical research, Neurology

## Abstract

Alzheimer's disease (AD) is a progressive disease, and the number of AD patients is increasing every year as the population ages. One of the pathophysiological mechanisms of AD is thought to be the effect of metabolomic abnormalities. There have been several studies of metabolomic abnormalities of AD, and new biomarkers are being investigated. Metabolomic studies have been attracting attention, and the aim of this study was to identify metabolomic biomarkers associated with AD and mild cognitive impairment (MCI). Of the 927 participants in the Nakayama Study conducted in Iyo City, Ehime Prefecture, 106 were selected for this study as Control (n = 40), MCI (n = 26), and AD (n = 40) groups, matched by age and sex. Metabolomic comparisons were made across the three groups. Then, correlations between metabolites and clinical symptoms were examined. The blood mRNA levels of the ornithine metabolic enzymes were also measured. Of the plasma metabolites, significant differences were found in ornithine, uracil, and lysine. Ornithine was significantly decreased in the AD group compared to the Control and MCI groups (Control vs. AD: 97.2 vs. 77.4; *P* = 0.01, MCI vs. AD: 92.5 vs. 77.4; *P* = 0.02). Uracil and lysine were also significantly decreased in the AD group compared to the Control group (uracil, Control vs. AD: 272 vs. 235; *P* = 0.04, lysine, Control vs. AD: 208 vs. 176; *P* = 0.03). In the total sample, the MMSE score was significantly correlated with lysine, ornithine, thymine, and uracil. The Barthel index score was significantly correlated with lysine. The instrumental activities of daily living (IADL) score were significantly correlated with lysine, betaine, creatine, and thymine. In the ornithine metabolism pathway, the spermine synthase mRNA level was significantly decreased in AD. Ornithine was decreased, and mRNA expressions related to its metabolism were changed in the AD group compared to the Control and MCI groups, suggesting an association between abnormal ornithine metabolism and AD. Increased betaine and decreased methionine may also have the potential to serve as markers of higher IADL in elderly persons. Plasma metabolites may be useful for predicting the progression of AD.

## Introduction

Alzheimer’s disease (AD) is a progressive disease, and the number of patients is increasing every year as the population ages. A variety of factors including genetic, epigenetic, and environmental factors, as well as aging, are thought to contribute to the disease, but none have as yet been clearly identified yet. Physical risk factors such as type 2 diabetes, obesity, hypertension, and hyperlipidemia are thought to potentially increase the risk of AD^1^. These risk factors are thought to be related to metabolic changes. Therefore, the study of blood metabolomics may show some underlying mechanisms associated with the pathophysiology of AD and may uncover new biomarkers and early therapeutic targets for AD^2,3^.

Blood-based biomarkers for AD are thought to reflect the pathology of the brain and are easier to collect than those in cerebrospinal fluid^4,5^. Metabolomics has attracted much attention^6,7^. Capillary electrophoresis-time-of-flight mass spectrometry (CE-TOFMS) is one of the comprehensive, quantitative, and high-throughput analytical tools used for metabolomics^8,9^. To date, CE-TOFMS has identified abnormal blood metabolites in patients with various neuropsychiatric diseases such as schizophrenia^10^, bipolar disorder^11^, major depressive disorder^12^, and autism spectrum disorder^13^. Preceding blood metabolomic studies of AD showed that the changes in some amino acids and polyamines, such as ornithine, might reflect the pathologies involved^14,15^. However, these changes in AD have not been consistently confirmed, and few studies have reported on mild cognitive impairment (MCI).

Thus, the purpose of this study was to profile metabolites in the blood of MCI, AD, and age-sex-matched cognitively normal controls (Control). Then, the correlations among clinical characteristics including cognitive function and activities of daily living (ADL) and the plasma levels of metabolites were examined. Finally, gene expression analyses of enzymes in candidate metabolic pathways were performed.

## Material and methods

### Subjects from the Nakayama Study

Nakayama Town is a rural community in Iyo City, Ehime Prefecture, Japan, with a population of 2655. The Nakayama Study was a population-based complete enumeration survey including all residents over 65 years of age in Nakayama Town from January 2017 through April 2018. Of the 1,512 residents, 927 were qualified for the study^16,17^, 40 subjects who met the criteria for AD and 26 who met the criteria for aMCI were selected out of 927 people, and then 40 healthy controls were randomly selected from remaining 861 subjects to be age and sex matched to the 40 AD subjects. All subjects or their families signed written, informed consent forms approved by the institutional ethics committees of Ehime University Graduate School of Medicine (Approval Number: 1610004). This study was performed in accordance with the Declaration of Helsinki. Basically, blood sampling was drawn after 2 h after eating, but 2 patients had their blood drawn before eating and 10 participant s had their blood drawn less than 2 h after eating. A self-administered questionnaire was used to collect data on education, smoking and drinking history, daily physical activity level, medical history, and medication use^16,17^. After data collection, the data were checked by trained interviewers. Height and weight were measured with light clothing, and the body mass index (BMI) was calculated from the measured values. Head magnetic resonance imaging (MRI) and the Mini Mental State Examination (MMSE) were also performed. All AD patients met the diagnostic criteria according to the National Institute on Aging/Alzheimer’s Association^18^, and a diagnosis of amnestic MCI was made for patients who satisfied the following criteria: (1) normal general cognitive function, with a Mini-Mental State Examination (MMSE) score ≥ 23; (2) on the delayed recall test of the Wechsler Memory Scale-Revised, the cutoff scores for any cognitive impairment were selected according to education status (≤ 8 points for 16 years of education, ≤ 4 points for 8–15 years, and ≤ 2 points for 0–7 years); (3) neuropsychiatric examination showing absence of dementia or depression as determined by geriatric neuropsychiatrists according to the Diagnostic and Statistical Manual of Mental Disorders, 3rd edition, revised (DSM-III R) (American Psychiatric Association, 1997) criteria; (4) no ADL impairment; and (5) hippocampal and parietal lobe atrophy without apparent cerebrovascular disease on brain MRI.

### Quantitative metabolome analysis

Blood sampling was performed during the participants’ visits for the Nakayama Study. Samples were sent from Ehime University to Tokushima University, where each 50-µL sample was mixed with 450 µL of methanol containing internal standards (10 µM) and vortexed. Chloroform (500 µL) and Milli-Q water (200 µL) were added, mixed thoroughly, then centrifuged (2300×*g*, 4 °C, 5 min). To remove macromolecules, 375 μL of the aqueous layer were filtered through a 5-kDa cutoff filter (EMD Millipore, Billerica, MA, USA). The filtrate was lyophilized and dissolved in 50 µL of Milli-Q water containing the reference compound before mass spectrometry analysis.

Metabolome measurements were conducted at Tokushima University. Plasma metabolite profiling and a mixture of 55 standard metabolites (50 µM each, HMT, Tsuruoka, Japan) were analyzed using a capillary electrophoresis electrospray ionization time-of-flight mass spectrometry (CE-ESI-TOFMS) system (Agilent 7100 CE - 6230 TOFMS, Agilent Technologies, Palo Alto, CA, USA). 55 cation mode metabolites with standard were measured. Then, the 35 metabolites were selected by counts (> 1000) and signal-to-noise ratio (> 3.0) of the peak area from the extracted ion chromatogram.

### Blood sample for mRNA expression

Total RNA was isolated from whole peripheral blood of the same participant in the metabolomic study using PaxGene Blood RNA Systems tubes (BD, Tokyo, Japan) according to the standard protocol. The RNA concentration and purity measured by NanoDrop-1000 (Thermo Fisher Scientific, Yokohama, Japan) showed a 260/280 ratio between 1.8 and 2.0. Then, cDNA was synthesized using RNA (1.0 μg) with the High-Capacity cDNA Reverse Transcription Kit (Applied Biosystems, Foster City, CA, USA). The specific TaqMan probes (Integrated DNA Technologies, Iowa, USA) were Hs.PT.39a.22214836 for glyceraldehyde-3-phosphate dehydrogenase (GAPDH), Hs.PT.56a.491276 for argininosuccinate lyase (ASL), Hs.PT.58.14740388 for nitric oxide synthase2 (NOS2), Hs.PT.56a.2920438 for argininosuccinate synthase1 (ASS1), Hs.PT.58.22990692.g for ornithine transcarbamylase (OTC), Hs.PT.56a.20779559 for arginase1 (ARG1), Hs.PT.58.2742899 for antizyme inhibitor 1 (AZIN1), Hs.PT.58.1971913 for ornithine decarboxylase antizyme1 (OAZ1), Hs.PT.58.26956824 for ornithine decarboxylase antizyme2 (OAZ2), Hs.PT.58.4341481 for ornithine decarboxylase antizyme3 (OAZ3), Hs.PT.58.27029915 for ornithine decarboxylase 1 (ODC1), and Hs.PT.58.15157347 for spermine synthase (SMS). Our previous studies have consistently identified GAPDH as a suitable housekeeping gene for blood gene expression analysis using the Paxgene blood RNA system^19,20^. The final volume of reactions was 10 µL with TaqMan Universal Master Mix (Applied Biosystems). Expression levels were examined in duplicate with a specific sample as a reference. The ΔΔCt method using StepOne software (Applied Biosystems) was used to measure the relative expression levels.

### Data analysis

Descriptive statistics were calculated for the Control, MCI, and AD groups. Normality of distributions was checked using the Shapiro–Wilk test. The mean difference among three groups was tested by one-way analysis of variance with Bonferroni’s multiple comparisons test or the Kruskal–Wallis test with Bonferroni’s multiple comparisons test according to normality. Spearman’s correlation test was used to determine the correlations among clinical parameters and each metabolite or mRNA level. All analyses were carried out using IBM® SPSS® Version 26. A two-tailed *P* < 0.05 was considered significant in all analyses.

## Results

### Participants’ characteristics

Study participants’ characteristics including age, sex, height, weight, BMI, MMSE, education, Barthel Index (BI), instrumental activities of daily living (IADL), geriatric depression scale 15 (GDS15), and medical history are presented in Table 1. In the table of subject characteristics (Table 1), patients with inadequate history taking were included: one subject with no Barthel Index, one subject with inadequate history taking in Diabetes, Hyperlipidemia, Drinking, and Smoking, respectively. The Control, MCI, and AD groups were selected so that they would be matched in sex, height, and weight. The MMSE scores were significantly decreased (Control vs. MCI vs. AD: 28.4 vs. 25.3 vs. 18.4, *P* < 0.01). The majority of the participants had finished junior high school. The BI was significantly lower in the AD group compared to the MCI and Control groups (Control vs. AD: 98.3 vs 93.6, *P* = 0.02, MCI vs. AD: 98.3 vs 93.6, *P* < 0.01). The same results were obtained for IADL (Control vs. AD: 11.5 vs 6.00, *P* < 0.01, MCI vs. AD: 11.2 vs. 6.00, *P* < 0.01). Of all participants, 37 (35.2%) had a history of drinking and 21 (20.0%) of smoking. The rates of hypertension, diabetes mellitus, and hyperlipidemia were 75 (70.8%), 19 (18.1%), and 34 (32.4%), respectively. Although the number with hyperlipidemia was significantly higher in the Control group compared to the AD group (20 vs, 6; *P* < 0.01). In the Participant characteristics (Blood test), significant results were obtained for ALT, but additional tests did not yield significant results (Table 2).

**Table 1:** Participant characteristics. One (AD) had no information on diabetes, hyperlipidemia, drinking and smoking.

	Control	MCI	AD	*P*
	40	26	40	
Age (SD) years	81.8 ± 2.26	82.4 ± 5.49	83.8 ± 4.79	0.14
Female (%)	26/40 (65.0)	17/26 (65.4)	26/40 (65.0)	0.99
Height (SD) cm	150 ± 8.10	149 ± 11.1	146 ± 7.87	0.15
Weight (SD) kg	54.1 ± 9.36	50.1 ± 8.68	49.0 ± 9.55	0.04
BMI (SD) kg/m^2^	24.0 ± 2.87	22.5 ± 2.91	22.9 ± 3.49	0.14
MMSE (SD)	28.4 ± 1.51	25.3 ± 1.66	18.4 ± 3.72	< 0.01
**Education (%)**				
≤ Finished primary school	1/40 (2.5)	3/26 (11.5)	6/40 (15.0)	
≤ Finished junior high school	25/40 (62.5)	12/26 (46.2)	26/40 (65.0)	
≤ Finished high school	13/40 (32.5)	10/26 (38.5)	8/40 (20.0)	
≤ Finished college, university	1/40 (2.5)	1/26 (3.8)	0/40 (0.0)	
Barthel Index (SD)	98.3 ± 5.13	98.3 ± 8.83	93.6 ± 10.8	< 0.01
IADL (SD)	11.5 ± 2.22	11.2 ± 3.25	6.00 ± 4.82	< 0.01
GDS15 (SD)	2.45 ± 2.01	3.08 ± 2.24	3.33 ± 2.97	0.45
**Medical history (%)**				
Hypertension (%)	28/40 (70.0)	18/26 (69.2)	31/40 (77.5)	0.68
Diabetes (%)	9/40 (22.5)	3/26 (11.5)	7/39 (17.9)	0.53
Hyperlipidemia (%)	20/40 (50.0)	9/26 (34.6)	6/39 (15.4)	< 0.01
Drinking (%)	16/40 (40.0)	10/26 (38.5)	11/39 (28.2)	0.51
Smoking (%)	8/40 (20.0)	5/26 (19.2)	8/39 (20.5)	0.99

*BMI* body mass index, *MMSE* Mini Mental State Examination, *IADL* instrumental activities of daily living, *GDS15* geriatric depression scale 15.

**Table 2:** Participant characteristics (Blood test).

	Control (N = 40)	MCI (N = 26)	AD (N = 40)	*P*
WBC (× 10^3^/μL)	6.08 ± 1.50	6.31 ± 3.94	5.84 ± 1.31	0.64
RBC (× 10^6^/μL)	4.32 ± 0.41	4.27 ± 0.65	4.24 ± 0.40	0.74
Hb (g/dL)	13.3 ± 1.19	13.0 ± 1.62	13.1 ± 1.32	0.69
Ht (%)	41.3 ± 3.55	40.7 ± 5.16	40.6 ± 3.89	0.71
MCV (fL)	95.6 ± 3.55	95.9 ± 7.06	96.0 ± 5.09	0.96
MCH (pg)	30.7 ± 1.34	30.7 ± 2.75	30.9 ± 2.03	0.93
MCHC (%)	32.1 ± 0.73	32.0 ± 1.13	32.2 ± 0.85	0.84
PLT (× 10^4^/μL)	23.3 ± 3.83	26.4 ± 20.2	23.2 ± 6.27	0.80
TP (g/dL)	7.35 ± 0.40	7.23 ± 0.46	7.24 ± 0.38	0.39
ALB (g/dL)	4.19 ± 0.30	4.08 ± 0.35	4.12 ± 0.31	0.39
Total-Bil (mg/dL)	0.77 ± 0.28	0.69 ± 0.23	0.70 ± 0.25	0.40
AST (U/L)	24.8 ± 5.96	26.2 ± 9.19	26.9 ± 14.1	0.73
ALT (U/L)	17.7 ± 7.34	18.5 ± 11.1	18.6 ± 29.0	0.04
ALP (U/L)	243 ± 75.4	264 ± 77.8	284 ± 201	0.32
LDH (U/L)	223 ± 33.6	237 ± 56.6	228 ± 31.9	0.76
γ-GT (U/L)	22.4 ± 11.5	21.6 ± 10.8	34.9 ± 78.7	0.80
CPK (U/L)	117 ± 43.5	121 ± 71.0	126 ± 92.3	0.66
Tchol (mg/dL)	194 ± 27.3	193 ± 38.0	192 ± 31.2	1.00
LDL (mg/dL)	108 ± 21.7	110 ± 28.3	110 ± 28.5	0.88
HDL (mg/dL)	58.2 ± 15.7	60.0 ± 15.9	57.1 ± 15.0	0.76
HbA1c (NGSP, %)	5.91 ± 0.68	5.66 ± 0.37	5.96 ± 1.00	0.44
GA (%)	16.3 ± 3.10	15.8 ± 1.96	17.4 ± 4.92	0.48
BUN (mg/dL)	17.8 ± 3.93	17.2 ± 5.90	17.0 ± 4.98	0.38
Cr (mg/dL)	0.72 ± 0.19	0.74 ± 0.37	0.74 ± 0.23	0.80
eGFR (mL/min/1.73 m^2^)	80.7 ± 11.3	81.5 ± 15.3	77.6 ± 14.1	0.66
UA (mg/dL)	4.82 ± 0.81	4.62 ± 1.32	4.70 ± 1.37	0.79
Na (mEq/L)	141 ± 2.05	141 ± 2.47	141 ± 2.49	0.40
K (mEq/L)	4.94 ± 0.70	5.06 ± 0.74	4.99 ± 0.45	0.76
Cl (mEq/L)	103 ± 2.21	103 ± 3.23	103 ± 2.93	0.67
TSH (μIU/mL)	2.97 ± 1.89	2.89 ± 2.21	2.37 ± 1.27	0.35
FT4 (ng/dL)	1.14 ± 0.19	1.07 ± 0.16	1.11 ± 0.13	0.49
CRP (mg/dL)	0.12 ± 0.14	0.07 ± 0.10	0.10 ± 0.12	0.14

### Comparison of plasma metabolites in the Control, MCI, and AD groups

**Figure 1:**
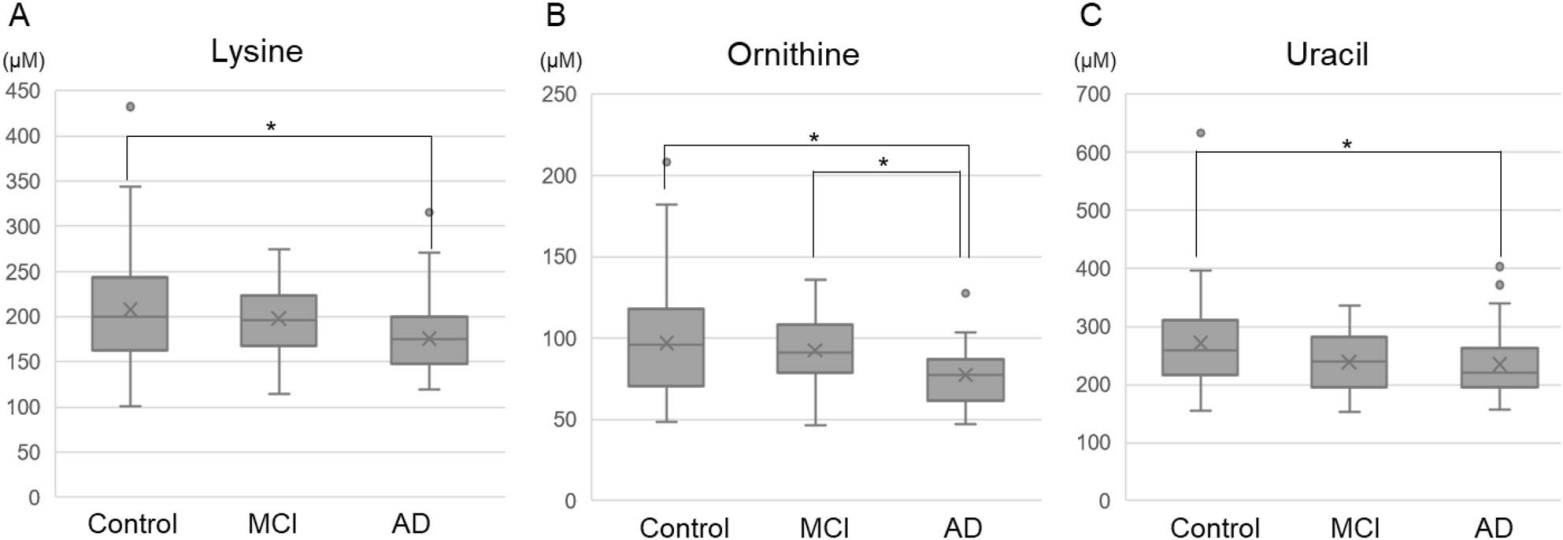
Comparisons of mean plasma amino acid values for (**A**) lysine, (**B**) ornithine, and (**C**) uracil. *P < 0.05.

In the plasma metabolite comparisons, 35 metabolites were examined. Significant differences were found only in ornithine, uracil, and lysine (Table 3, Fig. 1). For ornithine, there was a significant difference between the Control and AD groups and between the MCI and AD groups (Control vs. AD: 97.2 µM vs. 77.4 µM; *P* = 0.01, MCI vs. AD: 92.5 µM vs. 77.4 µM; *P* = 0.02). For uracil and lysine, there were significant differences between the Control and AD groups (uracil, Control vs. AD: 272 µM vs. 235 µM; *P* = 0.04; Lysine, Control vs. AD: 208 µM vs. 176 µM; *P* = 0.03).

**Table 3:** Mean values of plasma amino acids.

	Control (N = 40)	MCI (N = 26)	AD (N = 40)	*P*
Alanine (µM)	509 ± 90.0	504 ± 112	495 ± 109	0.82
Arginine (µM)	107 ± 25.0	105 ± 28.4	103 ± 26.7	0.81
Asparagine (µM)	59.7 ± 9.93	58.9 ± 12.0	56.9 ± 11.6	0.51
Aspartic acid (µM)	30.1 ± 8.72	30.4 ± 9.67	28.4 ± 14.8	0.11
Cysteine (µM)	10.3 ± 2.47	9.90 ± 2.72	9.99 ± 2.56	0.83
Glutamine (µM)	752 ± 91.6	725 ± 119	746 ± 109	0.29
Glutamic acid (µM)	113 ± 35.3	110 ± 49.1	104 ± 49.2	0.22
Glycine (µM)	288 ± 63.8	320 ± 115	287 ± 73.1	0.24
Histidine (µM)	88.3 ± 15.2	82.6 ± 15.1	80.8 ± 13.2	0.06
Isoleucine (µM)	63.6 ± 23.1	64.9 ± 17.5	59.6 ± 20.3	0.37
Leucine (µM)	118 ± 38.1	122 ± 29.6	110 ± 29.9	0.24
Lysine (µM)	208 ± 62.9	198 ± 45.6	176 ± 40.5	0.02
Methionine (µM)	23.0 ± 7.33	23.8 ± 7.01	24.8 ± 6.88	0.32
Phenylalanine (µM)	82.2 ± 14.5	78.0 ± 14.1	76.0 ± 16.6	0.13
Proline (µM)	233 ± 89.6	203 ± 47.8	223 ± 75.0	0.51
Serine (µM)	153 ± 30.1	156 ± 28.4	147 ± 32.1	0.36
Threonine (µM)	145 ± 34.3	130 ± 34.5	134 ± 37.2	0.19
Tryptophan (µM)	60.2 ± 13.0	57.9 ± 14.7	56.2 ± 11.9	0.40
Tyrosine (µM)	81.1 ± 18.6	75.4 ± 15.1	75.3 ± 23.8	0.23
Valine (µM)	243 ± 51.7	250 ± 48.4	244 ± 52.7	0.83
Anthranilic acid (µM)	1.12 ± 0.42	0.95 ± 0.23	0.99 ± 0.28	0.25
Betaine (µM)	72.1 ± 20.9	72.7 ± 18.1	68.3 ± 20.3	0.33
Choline (µM)	33.2 ± 6.75	33.3 ± 7.23	31.7 ± 6.68	0.45
Citrulline (µM)	40.6 ± 13.2	40.1 ± 17.5	37.5 ± 9.19	0.75
Creatine (µM)	49.9 ± 29.5	50.0 ± 25.9	52.5 ± 33.8	0.95
Creatinine (µM)	71.7 ± 17.5	71.9 ± 40.0	67.9 ± 23.5	0.21
GABA (µM)	4.23 ± 4.33	4.42 ± 10.3	3.38 ± 4.92	0.14
Hydroxyproline (µM)	13.6 ± 5.15	13.2 ± 5.80	13.2 ± 6.24	0.71
Hypoxanthine (µM)	16.2 ± 4.98	14.3 ± 4.63	15.0 ± 5.90	0.11
Kynurenine (µM)	2.22 ± 0.64	2.05 ± 0.63	1.97 ± 0.60	0.19
N,N-Dimethylglycine (µM)	4.37 ± 1.14	4.36 ± 1.26	4.62 ± 1.36	0.59
Ornithine (µM)	97.2 ± 34.1	92.5 ± 20.5	77.4 ± 17.7	< 0.01
Thymine (µM)	68.0 ± 22.2	71.4 ± 20.6	78.8 ± 30.0	0.07
Uracil (µM)	272 ± 82.9	239 ± 49.4	235 ± 59.1	0.04
Uridine (µM)	14.2 ± 4.25	14.5 ± 6.76	14.3 ± 5.47	0.95

### Relationships between metabolite relative concentrations and clinical symptoms

The relationships between the concentrations of metabolites and clinical symptoms evaluated by the MMSE, BI, IADL, and GDS15 were examined. In total samples, the MMSE score was significantly correlated with lysine (r = 0.23, *P* < 0.05), ornithine (r = 0.28, *P* < 0.01) and thymine (r = − 0.22, *P* < 0.05). The BI was significantly correlated with lysine (r = 0.21, *P* < 0.05). The IADL score was significantly correlated with lysine (r = 0.20, *P* < 0.05), betaine (r = 0.34, *P* < 0.01), creatine (r = − 0.25, *P* < 0.05), and thymine (r = − 0.27, *P* < 0.01). The GDS15 score was correlated with creatinine (r = − 0.23, *P* < 0.05).

### Comparison of mRNA expression levels of metabolic enzymes

**Figure 2:**
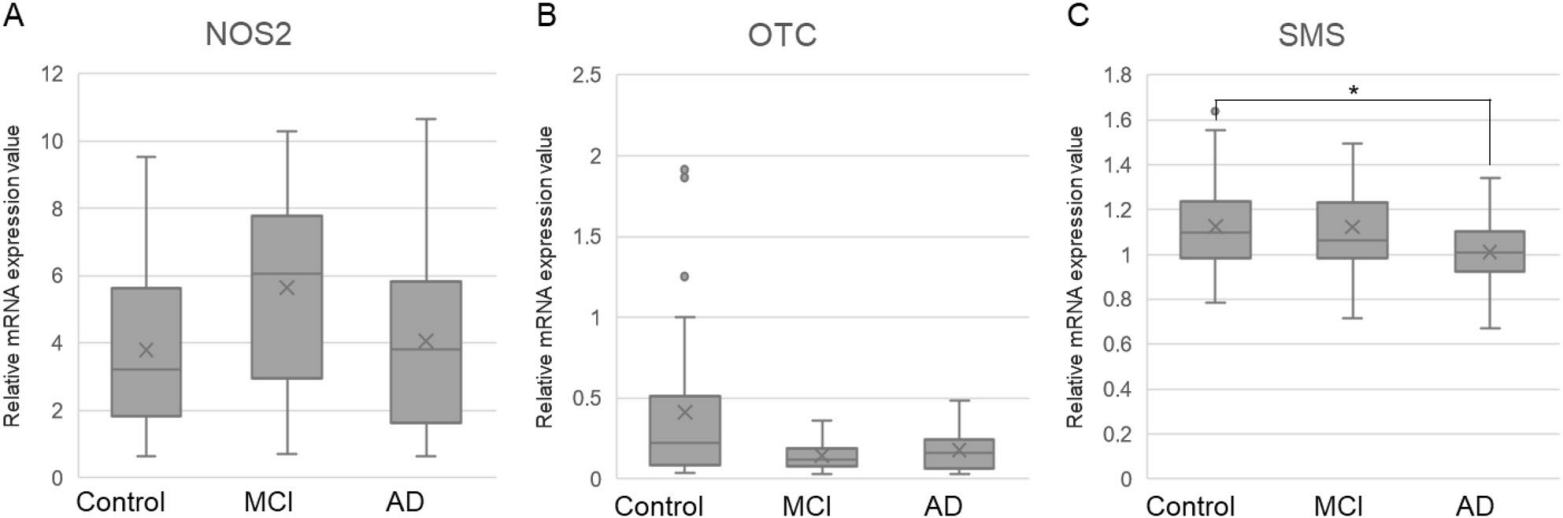
Comparisons of mRNA expression levels of metabolism enzymes for (**A**) NOS2, (**B**) OTC, and (**C**) SMS. *P < 0.05.

The mRNA expression levels of the metabolic enzymes in ornithine pathways were measured (Fig. 2). SMS, NOS2, and OTC mRNA levels were significantly different among the three groups (Table 4). On post hoc analysis, the SMS mRNA level was significantly lower in the AD group than in the Control group (Control vs. AD: 1.13 vs 1.01; *P* = 0.04). The NOS2 mRNA level was higher in the MCI group than in the Control group (Control vs. MCI: 3.80 vs 5.66; *P* = 0.06), whereas the OTC mRNA level was lower in the MCI group compared to the Control group (Control vs. MCI: 0.41 vs 0.14; *P* = 0.06).

**Table 4:** Relative mRNA expression levels of metabolism enzymes.

	N	Control	N	MCI	N	AD	*P*
ASL	37	1.39 ± 0.21	24	1.47 ± 0.24	40	1.35 ± 0.34	0.26
NOS2	33	3.80 ± 2.51	23	5.66 ± 2.85	32	4.06 ± 2.91	0.04
ASS1	37	0.48 ± 0.28	24	0.32 ± 0.10	37	0.36 ± 0.14	0.14
OTC	35	0.41 ± 0.49	25	0.14 ± 0.09	34	0.18 ± 0.13	0.04
ARG1	39	0.42 ± 0.27	26	0.41 ± 0.27	39	0.40 ± 0.28	0.83
AZIN1	37	1.17 ± 0.15	26	1.21 ± 0.19	38	1.18 ± 0.19	0.56
OAZ1	37	0.83 ± 0.35	24	0.80 ± 0.40	38	0.82 ± 0.42	0.64
OAZ2	37	1.29 ± 0.26	25	1.35 ± 0.32	38	1.25 ± 0.33	0.46
OAZ3	39	1.34 ± 0.65	25	1.63 ± 0.73	39	1.53 ± 0.69	0.17
ODC1	39	1.45 ± 0.46	25	1.47 ± 0.61	39	1.26 ± 0.39	0.12
SMS	38	1.13 ± 0.20	25	1.12 ± 0.21	38	1.01 ± 0.14	0.01

### Discriminant analysis of the AD and Control groups

Discriminant analysis was conducted with the levels of ornithine, lysine, and uracil (Wilks lambda = 0.82, *P* < 0.01). The discrimination score was calculated for each sample as follows:

$$ {\text{Discrimination}}\,{\text{score}} = 0.0{23} \times {\text{ornithine}} + 0.00{8} \times {\text{lysine}} + 0.00{6} \times {\text{uracil}} - {5}.{13} $$

**Figure 3:**
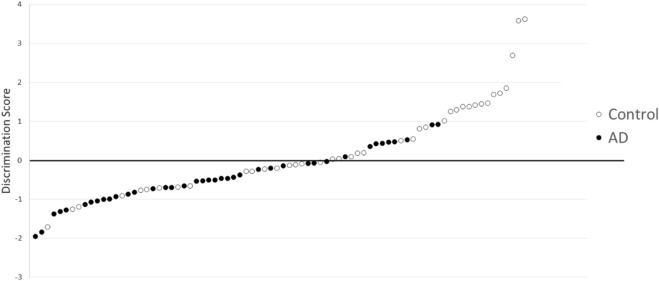
Discrimination score for three metabolites (○: Control, ●: AD). The distribution of scores, with sensitivity and specificity of 77.5% and 57.5%, respectively, in differentiating between healthy controls and AD patients.

The analysis demonstrated sensitivity and specificity of 77.5% and 57.5%, respectively (Fig. 3).

## Discussion

**Figure 4:**
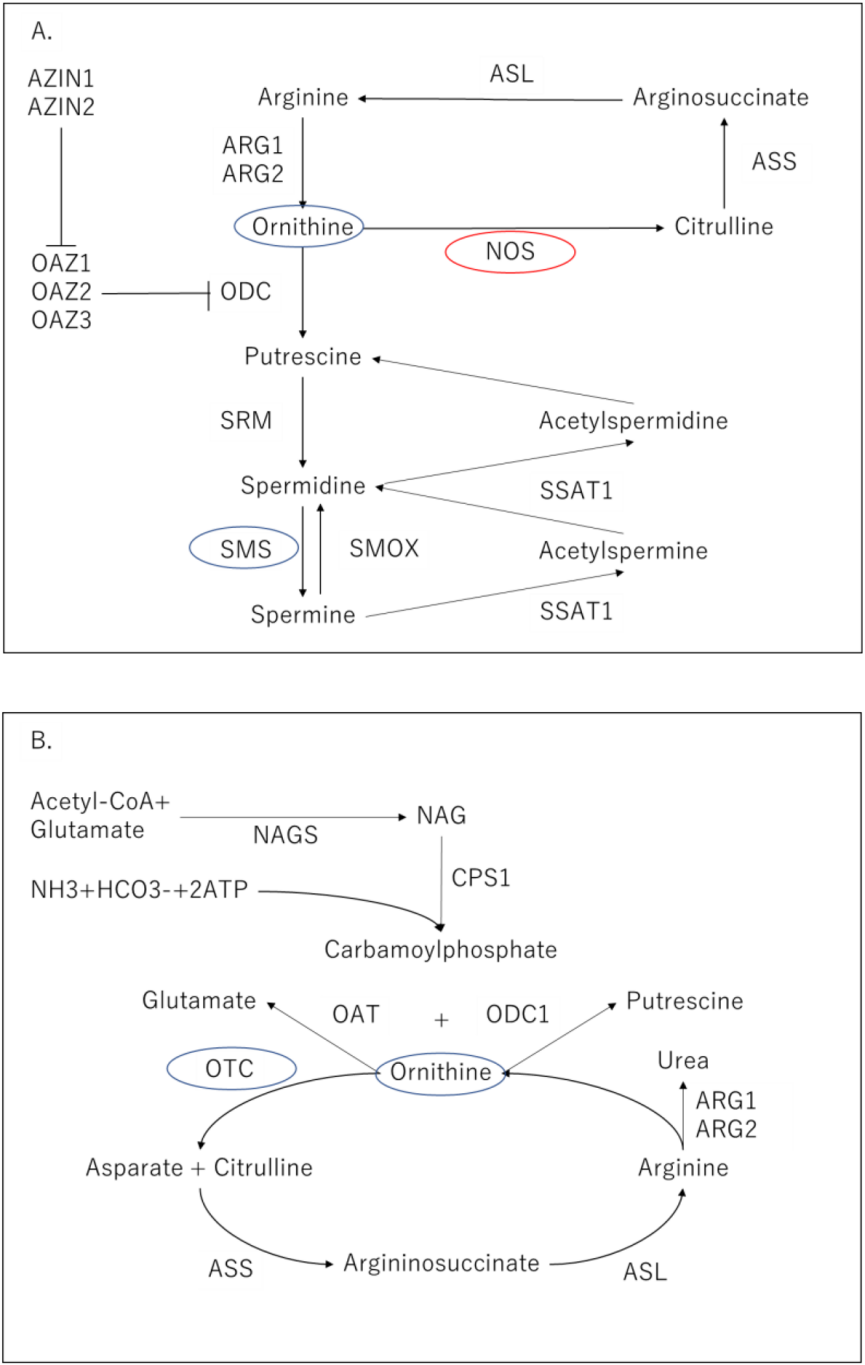
Polyamine pathway and urea cycle. (**A**) The polyamine pathway in mammalian cells (33,586,680 Fig. 1) ARG1: arginase1, *ARG2* arginase2, *ODC* ornithine decarboxylase, *SRM* spermidine synthase, *SMS* spermine synthase, *SMOX* spermine oxidase, *AZIN1* antizyme inhibitor 1, *OAZ1* ornithine decarboxylase antizyme 1, *SSAT1* spermidine/spermine N1-acetyltransferase 1, *NOS* nitric oxide synthase, *ASL* argininosuccinate lyase, *ASS* argininosuccinate synthase. (**B**) Urea cycle (31,978,055 Fig. 1) *NAG* N-acetyl glutamate, *NAGS* N-acetylglutamate synthase, *CPS1* carbamoyl phosphate synthetase I, *OAT* ornithine aminotransferase, ornithine aminotransferase, *ODC1* ornithine decarboxylase 1, *OTC* ornithine transcarbamylase.

In the present study, ornithine, uracil, and lysine were decreased in AD patients compared to healthy controls. The decrease of ornithine may be consistent with the previous study showing that ornithine was decreased in the CSF of AD patients, and that the polyamine pathway (and metabolites of the tryptophan-kynurenine pathway) was also downregulated^21^. In addition, it has been reported that decreased ornithine is associated with lower cognitive function in the general population^22^. As for ornithine metabolism pathways (Fig. 4), it has been shown that metabolites of arginine and polyamine are altered in the blood of MCI and AD patients^15^. However, AD patients have been reported to have decreased lipid metabolites such as HDL subfractions and DHA, and increased glutamine and ornithine levels^22^. Knockout mice that lack an ornithine degradation system develop fatal hyperammonemia, and ornithine is considered to be an essential amino acid for the urea cycle^23^. Similarly, in humans, ornithine accumulation has been shown to cause hyperornithinemia-hyperammonemia-homocitrullinuria syndrome. At present, the cause of this disorder is not clear, but clinically, it is believed to cause mental retardation^22^. The present results indicated that ornithine and polyamine pathways may be associated with cognitive function in AD. There has been no report showing significant changes in uracil and lysine in AD, and further studies of their involvement in the pathophysiology of AD are needed.

Interestingly, there was a significant positive correlation between betaine and IADL. Betaine is known to act against various biological stresses and serve as a substrate in the betaine-homocysteine S-methyltransferase (BHMT) reaction, converting homocysteine to methionine^24^. Betaine intake is known to improve cognitive impairment in both AD mice^25^ and AD patients^26^. Thus, high betaine levels may be associated with higher ADL in elderly persons.

We hypothesized that the decreased ornithine level might be due to an aberrant polyamine pathway or urea cycle (Fig. 4). Thus, mRNA levels of enzymes in the polyamine pathway and urea cycle were examined. In the polyamine pathway, the SMS mRNA level was significantly decreased in AD, and the NOS2 mRNA level was increased in MCI. In the urea cycle, the OTC mRNA level was decreased in MCI. There may be some correlations between the ornithine concentration and mRNAs related to the amino acid, but direct relationships among them could not be found.

The discriminant analysis between the AD and Control groups using the concentrations of 3 metabolites (ornithine, lysine, and uracil) demonstrated sensitivity and specificity of 77.5% and 57.5%, respectively. The relatively low specificity suggested that only three metabolites had not enough potential to use in the clinical setting for differentiating AD from healthy subjects, although there were significant relationships among these three metabolites and AD. Considering AD and MCI due to AD-related cognitive changes as a continuous spectrum, the intermediate discrimination score of the MCI group is reasonable (mean ± SD, AD − 0.53 ± 0.69 vs. MCI 0.02 ± 0.93 vs. Control 0.40 ± 1.22). Emerging evidence has suggested that biomarkers detecting progression of AD are needed for more personalized treatments, and blood metabolomics are among such novel candidates^27,28^.

This work’s strength was that it was a population-based, complete enumeration study, but it had some limitations. Since few studies have examined the relationships between metabolomics and neurodegenerative diseases, further studies are warranted to validate the current findings in other cohorts, both cross-sectionally and longitudinally. Further research is needed to diagnose other neurodegenerative diseases such as dementia with Lewy bodies. The significance level of 5% for metabolites was not adjusted for multiple testing, which may lead to type I error. Moreover, the sample size was relatively small. Due to the limited power of this study, the negative findings should not be interpreted as showing no associations.

## Conclusion

Ornithine was decreased and mRNA expressions related to its metabolism were changed in the AD group compared to the Control and MCI groups, suggesting an association between abnormal ornithine metabolism and AD. Increased betaine and decreased methionine may have the potential to serve as markers of better IADL in elderly persons. Our results suggest that plasma metabolomics may be useful for predicting the progression of AD.

### Supplementary Information

Supplementary Information 1.

Supplementary Information 2.

## Data Availability

All data generated or analyzed during this study are included in this published article and its supplementary information files.
