# Supplementary material for: Metabolomic alterations in the blood plasma of older adults with mild cognitive impairment and Alzheimer’s disease (from the Nakayama Study)
Source: Sci Rep. 2022 Sep 8;12:15205. doi: 10.1038/s41598-022-19670-y (PMC9458733; doi:10.1038/s41598-022-19670-y)
Supplement: Supplementary file 1 — Supplementary Information 1. [file 41598_2022_19670_MOESM1_ESM.pdf]

Supplement Table 1-1 #1. Logical memory results of WMS-R

	Ct	MCI	AD
Patients/All	4/40	26/26	36/40
Immediated recall test (Average Score ± SD)	8.50 ± 4.04	3.50 ± 2.76	1.75 ± 2.18
Delayed recall test (Average Score ± SD)	7.50 ± 1.73	2.08 ± 2.12	0.53 ± 1.28

Supplement Table 1-1 #2. Treatment status of hyperlipidemia, diabetes, and hypertension (number of persons)

		Ct	MCI	AD
Hypertension		28/40	18/26	31/40
	No treatment history	1	3	4
	Previous treatment	1	0	0
	Currently under treatment	24	15	24
	Treatment status unknown	2	0	3
Diabetes		9/40	3/26	7/39
	No treatment history	2	0	0
	Previous treatment	0	1	0
	Currently under treatment	6	0	7
	Treatment status unknown	1	2	0
Hyperlipidemia		20/40	9/26	6/39
	No treatment history	1	0	0
	Previous treatment	1	0	0
	Currently under treatment	18	9	6
	Treatment status unknown	0	0	0

Supplement Table 1-2. Correction for Bonferroni

		Mean Difference	SE	Standardization Mean difference	P-value	Adjusted P-value *
ALT						
	Ct-MCI	-0.26	7.73	-0.03	0.97	1
	Ct-AD	-15.7	6.86	-2.28	0.02	0.07
	MCI-AD	-15.4	7.73	-1.99	0.05	0.14

* Correction for Bonferroni

Supplemental Table 2. Post hoc Correction for Bonferroni test (Lysine, Ornithine, and Uracil)

		Mean Difference	SE	Standardization Mean difference	P-value	Adjusted P-value *
Lysine						
	Ct-MCI	-1.81	7.75	-0.23	0.82	1
	Ct-AD	-18.1	6.87	-2.63	0.01	**0.03**
	MCI-AD	-16.3	7.75	-2.1	0.04	0.11
Ornithine						
	Ct-MCI	-0.51	7.75	-0.07	0.95	1
	Ct-AD	-20.1	6.87	-2.92	0	**0.01**
	MCI-AD	-20.6	7.75	-2.66	0.01	**0.02**
Uracil						
	Ct-MCI	-11.9	7.75	-1.54	0.12	0.37
	Ct-AD	-17	6.87	-2.47	0.01	**0.04**
	MCI-AD	-5.01	7.75	-0.65	0.52	1

* Correction for Bonferroni

Supplement Table 3. Post hoc Correction for Bonferroni test (NOS2, OTC, and SMS)

		Mean Difference	SE	Standardization Mean difference	P-value	Adjusted P-value *
NOS2						
	Ct-MCI	-16.2	6.94	-2.33	0.02	0.06
	Ct-AD	-1.24	6.34	-0.2	0.84	1
	MCI-AD	-14.9	6.98	-2.14	0.03	0.1
OTC						
	Ct-MCI	-16.8	7.14	-2.36	0.02	0.06
	Ct-AD	-13.1	6.57	-1.99	0.05	0.14
	MCI-AD	-3.74	7.19	-0.52	0.6	1
SMS						
	Ct-MCI	-0.9	7.55	-0.12	0.91	1
	Ct-AD	-16.9	6.72	-2.51	0.01	**0.04**
	MCI-AD	-16	7.55	-2.12	0.03	0.1

* Correction for Bonferroni
